# Editorial Note: Calcein-effluxing human colon cancer cells are enriched for self-renewal capacity and depend on β-catenin

**DOI:** 10.18632/oncotarget.28903

**Published:** 2026-08-07

**Authors:** Joshua E. Allen, Wafik S. El-Deiry

**Affiliations:** ^1^Laboratory of Translational Oncology and Experimental Cancer Therapeutics, Department of Medicine (Hematology/Oncology), Penn State Hershey Cancer Institute; ^2^American Cancer Society, Atlanta, Georgia

The Oncotarget Scientific Integrity Office checked a data misplacement in the article: Allen JE, El-Deiry WS. Calcein-effluxing human colon cancer cells are enriched for self-renewal capacity and depend on β-catenin. Oncotarget. 2013 Feb;4(2):184-91. doi: 10.18632/oncotarget.883. PMID: 23468473. PMCID: PMC3712565.

Specifically, in Supplementary Figure 1A the gating images of HT29 cells for “Negative control” and “Calcein+CD133+CD26” as well as the plots for “Calcein” and “Calcein+Verapamil” are duplicates.

The Oncotarget Scientific Integrity Office contacted the authors to explain the data misplacement. The corresponding author Dr. Wafik S. El-Deiry acknowledged that errors may have occurred during the assembly of Supplementary Figure 1 but this error does not compromise article conclusions for two primary reasons. First, Supplementary Figure 1 presents exemplary plots (specifically gating, scatter plots, and histograms for analysis of CD133, CD26, and calcein populations) that were not used for the final calculations. Second, the data in this article is consistent with findings reported by Allen et al. (2009) [1], where the CloP was observed in the range of ~1–2% of the total population. The authors further noted that, due to the time elapsed since publication and multiple laboratory relocations (University of Pennsylvania in 2010, Penn State University in 2014, Fox Chase Cancer Center in 2019, and currently Brown University), the underlying raw data are no longer available.

The Oncotarget Scientific Integrity team has investigated the matter and found no additional issues. While we have concluded that this error does not affect the article’s overall conclusions, the identified issue cannot be corrected. Therefore, in accordance with COPE guidance, the journal is issuing this Editorial Note to inform our readers and maintain the transparency and integrity of the scientific record.

*COPE guidance: “Concerns raised by a complainant that do not warrant a correction or expression of concern could also be resolved by some other means (e.g., letter to the editor, or note or notification).” https://publicationethics.org/guidance/guideline/expressions-concern.

Original article: Oncotarget. 2012; 4:184–191. Calcein-effluxing human colon cancer cells are enriched for self-renewal capacity and depend on β-catenin. https://doi.org/10.18632/oncotarget.883
